# Metagenomic next-generation sequencing shotgun for the diagnosis of infection in connective tissue diseases: A retrospective study

**DOI:** 10.3389/fcimb.2022.865637

**Published:** 2022-12-08

**Authors:** Huyan Wang, Xiaowei Shi, Huanhuan Yang, Yan Du, Jing Xue

**Affiliations:** ^1^ Department of Rheumatology, the Second Affiliated Hospital of Zhejiang University School of Medicine, Hangzhou, China; ^2^ Department of Rheumatology and Immunology, Jinhua Hospital of Zhejiang University, Jinhua, China; ^3^ Department of Nephrology, Affiliated Hangzhou Xixi Hospital of Zhejiang University School of Medicine, Hangzhou, China

**Keywords:** metagenomic next-generation sequencing, infection, connective tissue diseases, immunosuppression, diagnosis

## Abstract

**Objective:**

Patients with connective tissue diseases (CTDs) are at high risk of infection due to various reasons. The purpose of the study was to investigate the infection diagnosis value of metagenomic next-generation sequencing (mNGS) shotgun in CTDs to guide the use of anti-infective therapy more quickly and accurately.

**Methods:**

In this retrospective study, a total of 103 patients with CTDs admitted with suspected infection between December 2018 and September 2021 were assessed using mNGS as well as conventional microbiological tests (CMT).

**Results:**

Among these 103 patients, 65 were confirmed to have an infection (Group I) and 38 had no infection (Group II). mNGS reached a sensitivity of 92.31% in diagnosing pathogens in Group I. Moreover, mNGS showed good performance in identifying mixed infection. In all infection types, lung infection was the most common. mNGS also played an important role in detecting *Pneumocystis jirovecii*, which was associated with low CD4+ T-cell counts inextricably.

**Conclusion:**

mNGS is a useful tool with outstanding diagnostic potential in identifying pathogens in patients with CTDs and conduce to provide guidance in clinical practice.

## Introduction

Connective tissue diseases (CTDs) have significant morbidity and mortality, due in part to concurrent infections (Falagas et al., 2007). The reasons why patients with CTDs show a peculiar vulnerability to infection are diverse: the changes in the intrinsic immune environment, the use of immunosuppressive drugs, and the severity of the disease and complications (Di Franco et al., 2017). One of the major immunological alterations is represented by impairment of the complement system, which is strongly associated to the development of systemic lupus erythematosus (SLE) and to an increased susceptibility to infection (Skattum et al., 2011). Since most patients with CTDs have autoimmune dysfunction, long-term immunosuppressive treatments are inevitable, such as glucocorticoid, conventional disease-modifying anti-rheumatic drugs, and biologics. Although therapeutic agents have improved the symptoms of patients substantially, they are also responsible for a wide spectrum of infections, especially when used in combination (Chiu and Chen, 2020). Infection can also be related to the organ damage due to rheumatic diseases. Some CTDs are more likely to be complicated with interstitial lung disease (ILD), such as systemic sclerosis and dermatomyositis (Kawano-Dourado and Lee, 2021). Thus, underlying lung lesions secondary to CTDs may increase the incidence of respiratory tract infections (Ricci et al., 2021).

It is necessary to identify the correct pathogens quickly, because inaccurate coverage and untimely treatment can give rise to side effects or fail to achieve the desired results. With respect to the diagnosis of bacterial or fungal infections, there are various diagnostic methods. Direct examination and culture are currently the most common procedures. Smear is convenient but the positive rate remained at a relatively low level. As culture is time-consuming, it may delay the optimal treatment time. Furthermore, not all kinds of pathogens are suitable to be cultured and pathogens infecting the immunocompromised host can also be difficult to grow. Moreover, empiric broad-spectrum antimicrobial therapy may contribute to the occurrence of false-negative culture results (Farrell et al., 2013). Viruses are usually tested by nucleic acid-based tests, serologic tests, and immunological tests, which lack specificity to some extent (Vemula et al., 2016). Therefore, there is an urgent need to address the problems of quick and accurate diagnosis.

Recently, it is becoming extremely difficult to ignore the existence of metagenomic next-generation sequencing (mNGS). As a novel technique that has high-throughput capabilities, is unbiased, is accurate, and has a rapid turnaround time, mNGS has proven invaluable for detecting pathogens in clinical samples (Chen et al., 2020). These advantages allow the mNGS test to specify multiple pathogens simultaneously and detect the exit of microbial even with low abundance. mNGS also makes it possible to identify difficult-to-culture microorganisms. Although some studies on the application of mNGS in infectious diseases have been carried out, no previous study has investigated its application in patients with CTDs suspected of infection. The purpose of this study is to evaluate the ability of mNGS to detect pathogens and analyze the characteristics of the distribution of pathogens in CTDs associated with infection.

## Methods

### Study design

In this retrospective study, we consecutively enrolled 103 patients with CTDs undergoing the mNGS test because of suspected infection who were admitted at the Department of Rheumatology, the Second Affiliated Hospital of Zhejiang University School of Medicine, China, from December 2018 to September 2021. Suspected infection was defined as patients who presented with symptoms, such as fever, cough, weakness, or radiographic involvement. Samples were obtained from sites of suspected infection as clinically indicated, including blood, sputum, joint fluid, bronchoalveolar lavage fluid (BALF), and tissue. Other conventional microbiological tests (CMT) were carried out according to the condition of each subject as clinically indicated, including Gram stain, acid-fast bacilli smear, bacterial culture, fungal smear and culture, T-SPOT.TB test, Epstein–Barr virus and Cytomegalovirus IgM and IgG antibody test, Epstein–Barr virus and Cytomegalovirus nucleic acid detection, (1,3)-β-D-glucan assays (G test), galactomannan assays (GM test), Widal agglutination test, *Cryptococcus neoformans* capsular polysaccharide antigen detection, *Aspergillus* IgG antibody detection, and respiratory microorganism IgM antibody test (*Mycoplasma pneumoniae*, *Chlamydia pneumoniae*, *Legionella pneumophila*, respiratory syncytial virus, influenza virus A, influenza virus B, parainfluenza virus, and adenovirus).

A retrospective-review protocol was approved by the Ethics Committee of the Second Affiliated Hospital of Zhejiang University School of Medicine in China [No. 2021 (1099)]. The ethics committee approved the waiver of informed consent due to the retrospective nature of the study. All research data were anonymously analyzed.

### Case definition

Two independent clinicians analyzed an anonymized data file for each patient. Data were collected for each patient including demographic data, types of CTDs, laboratory examinations, glucocorticoid and immunosuppressive treatments, and results of all microbiological tests.

In view of all available documentation, it was determined whether each patient was confirmed to have an infection (Group I) or an infectious etiology could be excluded (Group II). First, they determined the likelihood of an infectious origin implied by clinical symptoms. Then, they identified pathogens that were potentially responsible for the episode of infection, based on mNGS and other microbiological tests. For non-commonly reported pathogens, unless mNGS results were in accordance with the patient’s clinical features, the detected reads were classified as non-pathogenic microbe sequences. Last, an infection-related diagnosis was established.

### Sample processing

Low-speed centrifugation (1,500 *g* for 20 min) was performed to remove human cells in the samples including BALF, blood, and cerebrospinal fluid (CSF). For the blood, only the plasma was collected for further testing.

Samples were then homogenized using bead beating followed by DNA or RNA extraction using the IngeniGen DNA or RNA Extraction Kit (IngeniGen XMK Biotechnologies, Inc., Zhejiang, China). Briefly, a 300-μl sample was added to the lysate buffer and mixed thoroughly. The lysates were subsequently incubated for 30 min at 50°C. Then, magnetic beads were added to the mixture and placed stably for 10 min. The magnetic beads were washed with washing buffer 1 and washing buffer 2, respectively. Last, 60 μl of elution buffer, which had been preheated at 65°C to elute the nucleic acids off the beads, was added.

The DNA or RNA libraries were prepared using the IngeniGen DNA or RNA Library Prep Kit according to standard procedures. Briefly, the DNA was fragmented, and the Illumina-compatible adaptors were added to the fragmented DNA simultaneously by a tagment enzyme. The library was purified by magnetic beads and then amplified by 15 PCR cycles. DNase was used to remove residual human DNA and the RNA was fragmented, followed by double-strand cDNA synthesis, end repair, dA tailing, and adapter ligation.

Sequencing was performed on the Illumina MiniSeq (Illumina, San Diego, CA) using 2 × 75 bp chemistry. Sterile nuclease-free water as a negative control was included in each run to detect the background contaminants, involved in the process of nucleic acid extraction, library preparation, and sequencing. A kind of unique marine bacterium (100 CFU/ml) as an internal control was also added to each sample to monitor the whole process. Data analysis was performed using IngeniSeq MG, a proprietary automated shotgun metagenomics analysis platform for pathogen detection. Briefly, all raw reads were quality-filtered using an in-house made program, including filtering adapter contamination and low-quality and low-complexity reads. Next, the human host sequence mapped to the human reference genome (hg19) and other contaminant sequences that were known to be derived from the reagents were removed by using Burrows–Wheeler alignment, and the filtered sequences were de-duplicated and then matched against a curated database consisting of 20,343 microbial reference genomes, including 7,044 bacteria, 2,890 fungi, 9,233 virus, 172 parasites, 139 mycoplasma, and 128 chlamydia. All microbial reference genomes are downloaded from public databases, such as NCBI. The resulting hits were again filtered by a proprietary algorithm that further removed background contaminants that may appear during sampling processing and library preparation. The quality control matrix is briefly presented here: (1) a true-positive result is admitted only when the negative control has corresponding reads <10% compared with the sample; (2) the internal control should have reads >50 for the results to be valid in each sample.

### Data analysis

The Mann–Whitney *U* rank sum nonparametric test was utilized to compare the laboratory examination data of two groups. The Chi-square test was applied to assess the pathogen-specific diagnostic performance of mNGS, and kappa statistics were used to evaluate agreements between mNGS and CMT. *p*-values < 0.05 were considered statistically significant. Statistical analysis was performed using the SPSS statistical package 25.0 software. Figure drawings were accomplished by GraphPad Prism 8.0 software.

## Results

### General characteristics

In the present study, a total of 103 patients were enrolled. The baseline characteristics of the patients are listed in **Table 1**. Factors include gender, age, maximum body temperature before treatment, course of primary disease, and types of CTDs. Among the 103 patients, 65 were diagnosed with infection by clinical and microbiological evidence (Group I) and 38 were finally confirmed without infection (Group II). The laboratory examinations of the two groups are shown in **Table 2**, including white blood cell (WBC), neutrophil (N), lymphocyte (Ly), C-reactive protein (CRP), erythrocyte sedimentation rate (ESR), procalcitonin (PCT), ferritin, proportion and absolute CD4+ T-cell counts, and CD4+/CD8+ T-cell count ratio. Patients in Group I showed lymphopenia with a median lymphocyte count of 0.78 × 10^9^/L in peripheral blood and had significantly lower total T-cell proportion (*p* = 0.048), CD4+ T-cell counts (*p* < 0.001), and proportion (*p* = 0.007) compared with Group II, while PCT (*p* = 0.017) and ferritin (*p* < 0.001) were significantly increased compared to Group II.

**Table 1:** Demographics of the enrolled patients with suspected infection.

Characteristics	Group I (*n* = 65)	Group II (*n* = 38)	Overall (*n* = 103)
Gender, female	40 (61.5)	33 (86.8)	73 (68.9)
Age, years	58 (48–66)	55 (42–64.5)	57 (46–66)
Body temperature, °C	38.4 (37.4–39.3)	38.0 (37.2–39.2)	38.4 (37.4–39.2)
Primary disease course, months	12 (5–60)	24 (3.75–120)	18 (5–72)
CTDs			
Dermatomyositis/polymyositis	23	10	33
Rheumatoid arthritis	13	8	21
Systemic lupus erythematosus	11	7	18
Systemic vasculitis	5	5	10
Sjogren’s syndrome	3	3	6
Systemic sclerosis	4	0	5
IgG4-related disease	2	1	3
Adult-onset Still’s disease	2	1	3
Undifferentiated connective tissue disease	1	1	2
Sarcoidosis	0	1	1
Antiphospholipid antibody syndrome	1	0	1

Continuous variables are presented as median and interquartile range; binary variables are presented as number and percentage. Group I: patients were confirmed with infection. Group II: patients were confirmed without infection. CTDs, connective tissue diseases.

**Table 2:** Laboratory examinations of infection and non-infection patients.

Laboratory examination	Group I (*n* = 65)	Group II (*n* = 38)	*p*-value
WBC, ×10^9^/L	7.80 (5.05–10.60)	6.91 (4.55–11.75)	0.932
N, ×10^9^/L	6.74 (3.48–9.19)	4.64 (3.28–9.06)	0.573
Ly, ×10^9^/L	0.78 (0.48–1.22)	1.35 (0.89–1.83)	**<0.001**
CRP, mg/L	29.70 (11.20–79.90)	18.80 (7.68–51.40)	0.215
ESR, mm/h	48.00 (17.75–72.25)	63.00 (30.00–90.00)	0.072
PCT, ng/ml	0.25 (0.11–0.58)	0.12 (0.05–0.32)	**0.017**
Ferritin, μg/L	651.40 (293.70–1500.00)	183.20 (121.60–446.25)	**<0.001**
Total T-cell proportion, %	68.80 (55.10–79.36)	76.50 (66.23–81.78)	**0.048**
CD4+ T-cell counts,/mm^3^	201.16 (131.37–425.13)	492.11 (269.40–798.18)	**<0.001**
CD4+ T-cell proportion, %	33.10 (20.90–42.40)	42.50 (27.55–50.83)	**0.007**
CD8+ T-cell proportion, %	27.60 (22.35–43.75)	29.35 (20.3–37.43)	0.538
CD4+/CD8+ T-cell count ratio	1.16 (0.59–1.77)	1.32 (0.92–2.26)	0.089
Total B-cell proportion, %	11.10 (5.00–20.05)	12.70 (7.91–20.23)	0.486

Data are presented as median and interquartile. Group I: patients were confirmed with infection. Group II: patients were confirmed without infection. WBC, white blood cell; N, neutrophil; Ly, lymphocyte; CRP, C-reaction protein; ESR, erythrocyte sedimentation rate; PCT, procalcitonin. Bold text indicates a statistically significant difference.

### Distribution of clinically relevant pathogens

Sample sources of mNGS from all of the enrolled patients are shown in **Table 3**, including BALF, blood, and CSF. Among the 65 patients in Group I, distribution of infection sites is shown as follows. The most common site was lung (*n* = 54, 83.08%), followed by blood (*n* = 3, 4.62%), and skin and soft tissue (*n* = 3, 4.62%). Two patients with abdominal infection were diagnosed as subphrenic abscess and bacterial peritonitis. Two patients with brain infection were diagnosed as suppurative meningitis and viral encephalitis. One patient was diagnosed with infectious arthritis. Combined with CMT and mNGS, pathogens can be cleared in 61 patients but remain unknown in 4 patients. According to the results of pathogen distribution, bacteria (*n* = 27, 44.26%) was the most common pathogen identified, followed by fungi (*n* = 14, 22.95%) and virus (*n* = 6, 9.84%). Among the 27 patients infected by bacteria only, Gram-negative bacteria (*n* = 13, 20.00%) was the most frequent type, followed by Gram-positive bacteria (*n* = 8, 12.31%), and then *Mycobacteria* (*n* = 6, 9.23%). As for fungi, it should be noted that *Pneumocystis jirovecii* infected half of the patients. For those identified with *Mycobacteria*, three cases had *Mycobacterium tuberculosis* and there was one case each of *Mycobacterium avium*, *Mycobacterium chelonae*, and *Mycobacterium intracellulare*. Mixed infection was not rare and was found in 14 patients, 6 of whom were detected by mNGS only, 1 of whom by culture, and 7 of whom by both mNGS and conventional microbiological tests.

**Table 3:** Different sample sources from all of the enrolled patients.

Sample source	Overall (*n* = 103)
Bronchoalveolar lavage fluid	58
Blood	16
Lung and pleural biopsy	7
Joint fluid	5
Cerebrospinal fluid	4
Pleural effusion	4
Bone marrow	3
Skin and soft tissue	3
Pericardial fluid	1
Sputum	1
Ascites	1

### Performance of mNGS

**Figure 1:**
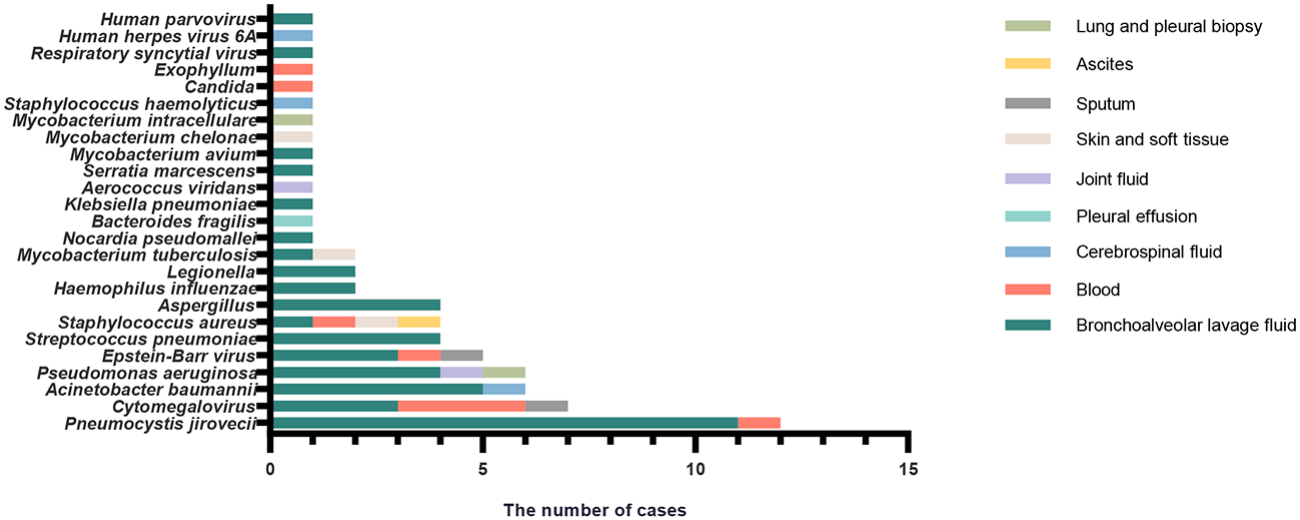
The correspondence between the sites of sample collection and frequency of different infection pathogens identified by mNGS in patients with confirmed infection (Group I).

The results of mNGS for the identification of clinically relevant pathogens are presented in **Figure 1**. The top four pathogens were *P. jirovecii* (*n* = 12), Cytomegalovirus (*n* = 7), *Acinetobacter baumannii* (*n* = 6), and *Pseudomonas aeruginosa* (*n* = 6). In seven subjects detected with Cytomegalovirus by mNGS, five were tested positive by PCR. Also worth noting was that Epstein–Barr virus was frequent; five subjects were positive for which by mNGS, and PCR confirmed the same results among four of them. **Figure 1** also revealed the relationship between the site of sample collection and its legitimate pathogen detected correspondingly. Herein, diagnostic sensitivity and specificity of mNGS were calculated using the clinical composite diagnosis as reference standard. As shown in **Table 4A**, mNGS showed a good sensitivity of 92.31%. The specificity of mNGS was 71.05%. NPV and PPV were 84.38% and 84.51%, respectively. The results of mNGS and the causative pathogens in the final diagnosis were concordant for 56 of the 65 patients for the analyses performed on samples in Group I, with a coincidence rate of 86.15%. The comparison of the diagnostic results of mNGS with the method of culture and CMT for 103 patients is shown in **Table 4B**. mNGS had an agreement rate of 42.7% with culture (*κ* = 0.102) and 54.4% with CMT (*κ* = 0.212). Additionally, mNGS increased the positive rate of diagnosis compared with CMT (*p* = 0.003). For nine subjects with discordance results in **Table 5**, mNGS reported five false-negative cases, three of whom were detected pathogens by other microbiological tests but the rest could not be identified by the pathogen yet. Among the remaining four subjects, mNGS results were different from other microbiological tests and were not considered as causative pathogens combined with clinical manifestations.

**Table 4a:** mNGS value for evaluating infection in CTDs patients.

Group	mNGS-positive	mNGS-negative
Confirmed infection	60	5
Excluded infection	11	27

mNGS, metagenomic next-generation sequencing. CTDs, connective tissue diseases. Sensitivity: 92.31%, specificity: 71.05%, positive predictive value (PPV): 84.38%, negative predictive value (NPV): 84.51%.

**Table 4b:** Diagnostic performance of mNGS compared with that of conventional methods.

Group	mNGS-positive	mNGS-negative	Agreement	*p*-value	Kappa
Culture-positive	13	1	42.7%	0.077	0.102
Culture-negative	58	31			
CMT-positive	27	3	54.4%	0.003	0.212
CMT-negative	44	29			

mNGS, metagenomic next-generation sequencing. CMT, conventional microbiologic tests, including testing mentioned in the study design section.

**Table 5:** Discordance results of different microbial detection tests in Group I.

Patient ID	Rheumatic disease	Infection clinical diagnosis	Sample	NGS result [reads]	Culture results	Other microbiological diagnostic testing results
29	Dermatomyositis	Pulmonary infection	BALF	*Haemophilus influenzae* [7513], *Moraxella catarrhalis* [169]	*Pseudomonas aeruginosa*	Sputum culture: *Aspergillus fumigatus*, G test (+), GM test (+)
30	Polymyositis	Pulmonary infection	BALF	Cytomegalovirus [92], Epstein–Barr virus [3]	*Staphylococcus aureus*	Negative
31	Polymyositis	Pulmonary infection	BALF	Negative	*Candida albicans*	Negative
32	Dermatomyositis	Pulmonary infection	BALF	Negative	Negative	Respiratory pathogen series antibodies: *Legionella pneumophila* IgM (+)
34	Systemic vasculitis	Pulmonary infection	Plasma	Negative	Negative	TSPOT (+)
48	Adult-onset Still’s disease	Pulmonary infection	Plasma	*Enterococcus casseliflavus* [46]	Negative	G test (+)
60	Undifferentiated connective tissue disease	Pulmonary infection	BALF	Negative	Negative	Sputum culture: *Burkholderia cepacia*
61	Sjögren’s syndrome	Pulmonary infection	Plasma	Negative	Negative	Sputum culture: *Candida albicans*, G test (+)
62	Overlap syndrome	Pulmonary infection	BALF	*Prevotella melaninogenica* [202397], *Veillonella parvula* [25988], *Rothia mucilaginosa* [25152], Epstein–Barr virus [48], Human betaherpesvirus 7 [9], Human betaherpesvirus *6B* [4]	Negative	G test (+)

BALF, bronchoalveolar lavage fluid.

For the 38 cases with no evidence of infection in Group II, mNGS detected pathogens in specimens from 11 patients, as shown in **Supplementary Table 1**. The microbes identified in the 11 cases by mNGS were present at relatively low abundance or considered as colonizing bacteria.

### Lymphocyte subsets and their association with mNGS results

**Figure 2:**
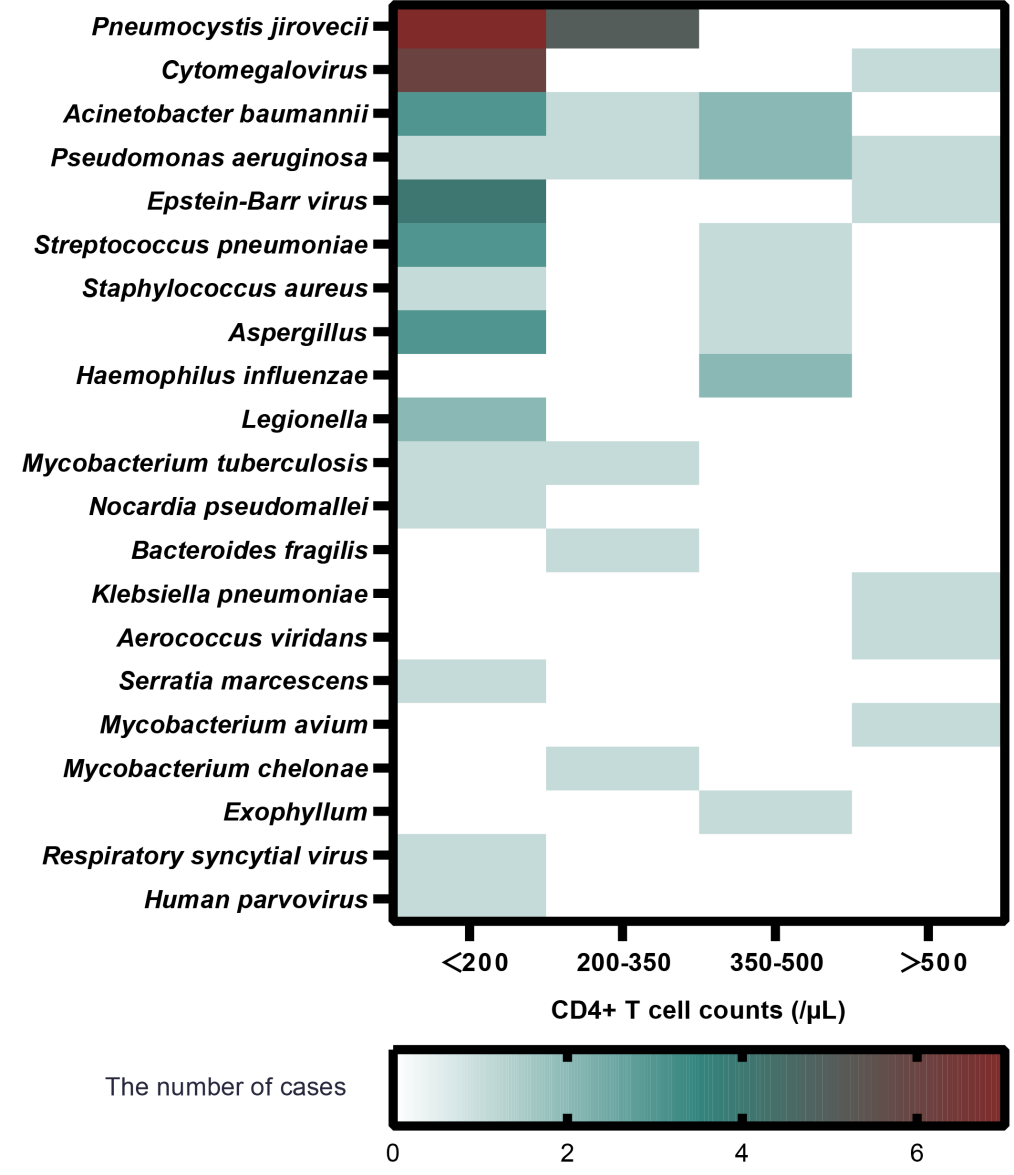
The relationship between pathogen type, infection frequency, and absolute value of CD4+ T-cell in patients with confirmed infection (Group I).

The absolute value of CD4+ T lymphocyte counts indicates the strength of immune function. CD4+ T-cell counts > 500/µl are generally considered as normal immune function. A cutoff of 200 CD4+ T cells/µl is defined as the key threshold to predict immune deficiency as usual (Freiwald et al., 2020). We investigated the pathogen type and frequency identified by mNGS with different degrees of CD4+ T-cell counts as in a prior study (Bordoni et al., 2019). Analysis of the subgroups based on the CD4+ T-cell counts is shown in **Figure 2**. In the CD4+ T cells<200/µl subgroup, *P. jirovecii* (*n* = 7) was the most detected pathogen, followed by Cytomegalovirus (*n* = 6).

## Discussion

Almost all patients with CTDs inevitably use immunosuppressive drugs to control the disease activity, which greatly increases the risk of infection (Wolfe and Peacock, 2017). Precise and timely microbial diagnosis of infection is essential for the clinical care of those immunocompromised patients. Some previous studies have reported about the mNGS diagnostic performance of infection in immunocompromised patients (Parize et al., 2017; Jiang et al., 2021). To our knowledge, this is the first study to evaluate the potential clinical relevance of mNGS involving different sites of specimen for the investigation of infection in a cohort of CTDs.

In our study, mNGS has a high sensitivity in identifying microorganisms and detecting a wide range of pathogens commonly and infrequently. This demonstrated that our study was capable of reflecting a variety of types of infectious diseases in adults admitted with suspected infection in a rheumatology unit. In the confirmed infection group (Group I), there were 19 patients suffering from dermatomyositis (DM)/polymyositis (PM), which ranked first in the enrolled CTDs. Six patients had anti-synthase antibody syndrome and five patients had anti-MDA5 positive dermatomyositis. A study confirmed the high mortality rate in Chinese DM/PM patients, with infection as the leading cause of death (Yang et al., 2020). Therefore, it is recommended to perform mNGS detection if we highly suspect infection in DM/PM patients.

It is noteworthy that for samples from 14 patients diagnosed with mixed infections, mNGS was able to detect pathogens in 13 of them. The diagnostic accuracy of mNGS was 92.86%. Wang et al. in their study found that the sensitivity of diagnosing mixed pulmonary infection by conventional diagnostic testing was 13.9%, which suggests that mixed infection is hard to detect (Wang et al., 2019). We suggest the timely use of mNGS when mixed infection or rare pathogens are suspected.

Lung infection was the most common in our study (54/65), identified by different samples. Forty-two were from BALF, seven from blood, two from lung and pleural biopsy, two from pleural effusion, and one from sputum. Clinicians tend to send BALF for examination, because it is generally accepted that BALF is the most direct sample to confirm whether patients suffer from lung infection (Zhang et al., 2014). In CTD-ILD, BAL has major clinical utility in excluding infections and in the diagnosis of specific patterns of lung damage. A retrospective study reviewing patients with DM-ILD undergoing bronchoscopy demonstrated that BAL can increase the diagnosis of infection in DM-ILD, and 22.1% patients commenced or changed antibiotic therapy on the basis of bronchoscopy results (He et al., 2021). Richter et al. found that pathogens were commonly grown from BALF of patients with granulomatosis polyangiitis (GPA) compared to those with idiopathic pulmonary fibrosis, and *Staphylococcus aureus* was particularly associated with patients with GPA (Richter et al., 2009). Bronchoscopy is a safe procedure in the general population, but as an invasive operation, there is no guarantee that it can be completely free from complications; serious adverse events could sometimes be observed in critically ill patients (Tomassetti et al., 2021). Thus, when patients in critical condition cannot tolerate bronchoscopy, it is more convenient to use blood samples. If it is possible, it will make great sense to design a prospective study to collect BALF and blood samples simultaneously to compare the accuracy of mNGS in identifying microorganisms in the future.

Our study clearly demonstrated that mNGS showed great potential to identify *P. jirovecii*. Similarly, Jiang et al. also reported that mNGS reached a sensitivity of 100% in diagnosing *P. jirovecii* pneumonia (PJP), which was remarkably higher than Gomori methenamine silver staining in non-HIV-infected patients (Jiang et al., 2021). In our study, mNGS figured out 12 cases with *P. jirovecii* infection, of which four subjects tested positive and eight subjects tested negative by G test. The diagnosis of PJP was based on the results of mNGS, combined with imaging manifestations in pulmonary and clinical symptoms such as decreasing oxygenation and dyspnea. Furthermore, subgroup analysis of CD4+ T-cell counts indicated that patients with low lymphocytes were vulnerable to *P. jirovecii* infection. Low CD4+ T-cell counts, attributable to glucocorticoid and immunosuppressant exposure to a certain extent, are identified as a significant risk factor for higher infection rate (Guillen et al., 2019). PJP is an opportunistic infection with high mortality among patients with underlying CTD conditions (Tadros et al., 2017). However, *P. jirovecii* cannot be cultured, and the sensitivity of conventional staining is low (Procop et al., 2004). Hence, the appearance of mNGS is utterly meaningful to detect *P. jirovecii*, especially on the condition of low CD4+ T-cell counts.

We can see that the rate of false-positive results had been high in patients who were confirmed without infection. Contamination is a probable factor, because of the various sample types, and the lack of a standard sample collecting method and site could affect the mNGS results. In addition, we can find that some of the microorganisms are the normal flora of the human oral cavity and upper respiratory tract, which are considered to be human symbiotic bacteria or colonizing bacteria, such as *Prevotella melaninogenica* and *Rothia mucilaginosa*. It is difficult to distinguish colonized pathogens from infection because there are no widely accepted quantitative cutoffs or threshold values for mNGS. Therefore, the definite diagnosis must be based on the comprehensive analysis of clinical characteristics, laboratory examinations, radiologic findings, and other microbiologic proofs rather than mNGS alone.

It should be mentioned that a recent Chinese study has attracted our attention (Su et al., 2022) because it was partially similar to our study. What separates them from us is that they only analyzed whole blood while our samples were collected from diverse sites and BALF was predominant. Types of CTDs with the highest incidence of infection were also slightly different; SLE and DM/PM ranked first and second in their results, whereas DM/PM and rheumatoid arthritis were more common in our study. Moreover, in their study, virus was the most common infection type, with Cytomegalovirus and Epstein–Barr virus being the highest, which showed a discrepancy with our findings, since our data showed that fungus such as *P. jirovecii* was observed the most. Distinctively, the pathogens of mixed infection were further classified in their results. Above all, both our results agree that mNGS had a higher detection rate than CMT.

This study had limitations. First, this was a retrospective study in a single center and the sample size was limited, which might have affected the accuracy of the evaluation of mNGS. Second, a negative control population of healthy individuals was not included in this study. Third, due to the complexity of infection, the sources of mNGS samples collected might not correspond to the infection site finally found through other microbial detection methods in a few patients. It may lead to a slight deviation in the results because we excluded these cases in advance during the study. Most importantly, we did not validate the diagnosis of some specific pathogens by quantitative real-time PCR (qPCR), such as *P. jirovecii, Mycobacterium tuberculosis*, and *Aspergillus*. qPCR as a molecular diagnostic method is applied widely and is highly sensitive for the analysis of species and abundance of microorganisms. However, because our study is a retrospective analysis, it would not be possible to verify the diagnosis of those samples by qPCR. We are convinced that it is of significance to conduct a comparison of the diagnostic efficiency of mNGS with qPCR in future research. To further evaluate the application of mNGS in the diagnosis of infections, multicenter prospective studies with a larger number of participants are encouraged. In addition, the sample collection method on mNGS performance needs further improvement.

## Conclusion

Although there are some limitations, our study indicated that mNGS has opened a new avenue for its clinical application in terms of detection of infectious pathogens for patients with CTDs. It has a high sensitivity in identifying microorganisms, especially *P.* jirovecii, and outperforms other methods in detecting mixed infections.

## Data Availability

The datasets presented in this study can be found in online repositories. The names of the repository/repositories and accession number(s) can be found below: China National Genebank, CNP0003551.

## References

[B1] Bordoni V. Brando B. Piselli P. Forini O. Perna F. E. Atripaldi U.. (2019). Naive/Effector CD4 T cell ratio as a useful predictive marker of immune reconstitution in late presenter HIV patients: A multicenter study. PloS One 14 (12), e0225415. doi: 10.1371/journal.pone.0225415 31869342 PMC6927630

[B2] Chen P. Sun W. He Y. (2020). Comparison of the next-generation sequencing (NGS) technology with culture methods in the diagnosis of bacterial and fungal infections. J. Thorac. Dis. 12 (9), 4924–4929. doi: 10.21037/jtd-20-930 33145066 PMC7578456

[B3] Chiu Y. M. Chen D. Y. (2020). Infection risk in patients undergoing treatment for inflammatory arthritis: non-biologics versus biologics. Expert Rev. Clin. Immunol. 16 (2), 207–228. doi: 10.1080/1744666X.2019.1705785 31852268

[B4] Di Franco M. Lucchino B. Spaziante M. Iannuccelli C. Valesini G. Iaiani G. (2017). Lung infections in systemic rheumatic disease: Focus on opportunistic infections. Int. J. Mol. Sci. 18 (2), 293. doi: 10.3390/ijms18020293 28146077 PMC5343829

[B5] Falagas M. E. Manta K. G. Betsi G. I. Pappas G. (2007). Infection-related morbidity and mortality in patients with connective tissue diseases: a systematic review. Clin. Rheumatol 26 (5), 663–670. doi: 10.1007/s10067-006-0441-9 17186117

[B6] Farrell J. J. Sampath R. Ecker D. J. Bonomo R. A. (2013). "Salvage microbiology": detection of bacteria directly from clinical specimens following initiation of antimicrobial treatment. PloS One 8 (6), e66349. doi: 10.1371/journal.pone.0066349 23825537 PMC3692526

[B7] Freiwald T. Buttner S. Cheru N. T. Avaniadi D. Martin S. S. Stephan C.. (2020). CD4(+) T cell lymphopenia predicts mortality from pneumocystis pneumonia in kidney transplant patients. Clin. Transplant. 34 (9), e13877. doi: 10.1111/ctr.13877 32277846

[B8] Guillen Y. Noguera-Julian M. Rivera J. Casadella M. Zevin A. S. Rocafort M.. (2019). Low nadir CD4+ T-cell counts predict gut dysbiosis in HIV-1 infection. Mucosal Immunol. 12 (1), 232–246. doi: 10.1038/s41385-018-0083-7 30171206

[B9] He L. Ge Y. Li S. Huang K. Liu X. Chen F.. (2021). Clinical role of bronchoalveolar lavage in dermatomyositis-associated interstitial lung disease. Rheumatol. (Oxford) 61 (1), 345–354. doi: 10.1093/rheumatology/keab586 34297087

[B10] Jiang J. Bai L. Yang W. Peng W. An J. Wu Y.. (2021). Metagenomic next-generation sequencing for the diagnosis of pneumocystis jirovecii pneumonia in non-HIV-Infected patients: A retrospective study. Infect. Dis. Ther. 10 (3), 1733–1745. doi: 10.1007/s40121-021-00482-y 34244957 PMC8322252

[B11] Kawano-Dourado L. Lee J. S. (2021). Management of connective tissue disease-associated interstitial lung disease. Clin. Chest Med. 42 (2), 295–310. doi: 10.1016/j.ccm.2021.03.010 34024405

[B12] Parize P. Muth E. Richaud C. Gratigny M. Pilmis B. Lamamy A.. (2017). Untargeted next-generation sequencing-based first-line diagnosis of infection in immunocompromised adults: a multicentre, blinded, prospective study. Clin. Microbiol. Infect. 23 (8), 574 e571–574 e576. doi: 10.1016/j.cmi.2017.02.006 28192237

[B13] Procop G. W. Haddad S. Quinn J. Wilson M. L. Henshaw N. G. Reller L. B.. (2004). Detection of pneumocystis jiroveci in respiratory specimens by four staining methods. J. Clin. Microbiol. 42 (7), 3333–3335. doi: 10.1128/JCM.42.7.3333-3335.2004 15243109 PMC446244

[B14] Ricci A. Pagliuca A. Vermi M. Pizzirusso D. Innammorato M. Sglavo R.. (2021). The role of lung colonization in connective tissue disease-associated interstitial lung disease. Microorganisms 9 (5), 932. doi: 10.3390/microorganisms9050932 33925354 PMC8146539

[B15] Richter A. G. Stockley R. A. Harper L. Thickett D. R. (2009). Pulmonary infection in wegener granulomatosis and idiopathic pulmonary fibrosis. Thorax 64 (8), 692–697. doi: 10.1136/thx.2008.110445 19359270

[B16] Skattum L. van Deuren M. van der Poll T. Truedsson L. (2011). Complement deficiency states and associated infections. Mol. Immunol. 48 (14), 1643–1655. doi: 10.1016/j.molimm.2011.05.001 21624663

[B17] Su R. Yan H. Li N. Ding T. Li B. Xie Y.. (2022). Application value of blood metagenomic next-generation sequencing in patients with connective tissue diseases. Front. Immunol. 13. doi: 10.3389/fimmu.2022.939057 PMC9376218 35979346

[B18] Tadros S. Teichtahl A. J. Ciciriello S. Wicks I. P. (2017). Pneumocystis jirovecii pneumonia in systemic autoimmune rheumatic disease: A case-control study. Semin. Arthritis Rheum 46 (6), 804–809. doi: 10.1016/j.semarthrit.2016.09.009 27814896

[B19] Tomassetti S. Colby T. V. Wells A. U. Poletti V. Costabel U. Matucci-Cerinic M. (2021). Bronchoalveolar lavage and lung biopsy in connective tissue diseases, to do or not to do? Ther. Adv. Musculoskelet Dis. 13, 1759720X211059605. doi: 10.1177/1759720X211059605 PMC8664307 34900002

[B20] Vemula S. V. Zhao J. Liu J. Wang X. Biswas S. Hewlett I. (2016). Current approaches for diagnosis of influenza virus infections in humans. Viruses 8 (4), 96. doi: 10.3390/v8040096 27077877 PMC4848591

[B21] Wang J. Han Y. Feng J. (2019). Metagenomic next-generation sequencing for mixed pulmonary infection diagnosis. BMC Pulm Med. 19 (1), 252. doi: 10.1186/s12890-019-1022-4 31856779 PMC6921575

[B22] Wolfe R. M. Peacock J. E. Jr. (2017). Pneumocystis pneumonia and the rheumatologist: Which patients are At risk and how can PCP be prevented? Curr. Rheumatol Rep. 19 (6), 35. doi: 10.1007/s11926-017-0664-6 28488228

[B23] Yang X. Hao Y. Zhang X. Geng Y. Ji L. Li G.. (2020). Mortality of Chinese patients with polymyositis and dermatomyositis. Clin. Rheumatol 39 (5), 1569–1579. doi: 10.1007/s10067-019-04910-w 31902027

[B24] Zhang Y. Chen Y. Chen Z. Zhou Y. Sheng Y. Xu D.. (2014). Effects of bronchoalveolar lavage on refractory mycoplasma pneumoniae pneumonia. Respir. Care 59 (9), 1433–1439. doi: 10.4187/respcare.03032 24962224

