# Supplementary material for: Metagenomic next-generation sequencing shotgun for the diagnosis of infection in connective tissue diseases: A retrospective study
Source: Front Cell Infect Microbiol. 2022 Dec 8;12:865637. doi: 10.3389/fcimb.2022.865637 (PMC9772835; doi:10.3389/fcimb.2022.865637)
Supplement: Supplementary file 1 [file Table_1.docx]

**Supplementary Table 1** False-positive results of mNGS in Group II.

| Patient ID | Rheumatic disease | Specimen | NGS result [reads] | Other microbiological diagnostic testing results | Possible reasons for being excluded infection |
| --- | --- | --- | --- | --- | --- |
| 66 | Undifferentiated connective tissue disease | Plasma | *Mycobacterium chelonae* [4] | Negative | Considered the mNGS result being contaminated. |
| 67 | Systemic lupus erythematosus | Cerebrospinal fluid | *Enterococcus casseliflavus* [50] | Negative | Considered the mNGS result being contaminated. Mental symptoms were considered to be caused by neuropsychiatric lupus rythematosus. |
| 69 | Dermatomyositis | Lung and pleural biopsy | *Stenotrophomonas maltophilia* [2519] | Negative | Infection was excluded by lung biopsy and mNGS. |
| 87 | Adult-onset Still's disease | BALF | Cytomegalovirus [5] | Negative | Considered the mNGS result being contaminated. Body temperature gradually returned to normal level after using glucocorticoid，the reason for fever was caused by the primary disease. |
| 90 | Dermatomyositis | BALF | *Haemophilus* [5] | Negative | Considered the mNGS result being contaminated. |
| 91 | Dermatomyositis | BALF | *Prevotella melaninogenica* [51586], *Neisseria meningitidis* [7406], *Veillonella parvula* [5671], *Rothia mucilaginosa* [5325], Human betaherpesvirus 7 [6] | Negative | Considered the mNGS result being contaminated. The symptoms of cough and sputum are considered to be caused by interstitial pneumonia. |
| 96 | Rheumatoid arthritis | Joint fluid | *Klebsiella pneumoniae* [49] | Negative | Considered the mNGS result being contaminated. |
| 97 | Sjögren's syndrome | BALF | *Pneumocystis jirovecii* [23] | Negative | The mNGS result was considered as oral colonizing bacteria. The symptoms of cough and sputum are considered to be caused by interstitial pneumonia. |
| 99 | Dermatomyositis | BALF | *Acinetobacter baumannii* [58], *Klebsiella pneumoniae* [19], *Pseudomonas aeruginosa* [14] | Negative | Considered the mNGS result being contaminated. The symptoms of chest tightness and shortness of breath are considered to be caused by interstitial pneumonia. |
| 100 | Dermatomyositis | BALF | *Pneumocystis jirovecii* [18] | Sputum culture Gomori methenamine silver staining：*Pneumocystis jirovecii* | The mNGS result was considered as oral colonizing bacteria. |
| 101 | Rheumatoid arthritis | BALF | *Pseudomonas aeruginosa* [6] | Negative | Considered the mNGS result being contaminated. |

BALF: bronchoalveolar lavage fluid.
